# Effects of neighborhood socioeconomic status on blood pressure in older adults

**DOI:** 10.1590/S1518-8787.2016050006595

**Published:** 2016-11-24

**Authors:** Katia Jakovljevic Pudla Wagner, Antonio Fernando Boing, SV Subramanian, Doroteia Aparecida Höfelmann, Eleonora D’Orsi

**Affiliations:** I Programa de Pós-Graduação em Saúde Pública. Universidade Federal de Santa Catarina. Florianópolis, SC, Brasil; II Department of Society, Human Development and Health. Harvard School of Public Health. Massachusetts, United States

**Keywords:** Aged, Hypertension, epidemiology, Risk Factors, Socioeconomic Factors, Cross-Sectional Studies

## Abstract

**OBJECTIVE:**

To test if the neighborhood socioeconomic status is associated with systolic blood pressure and hypertension in older adults.

**METHODS:**

A cross-sectional population-based study with a sample of 1,705 older adults from Florianópolis, SC, Southern Brazil. The contextual variable used was the average years of schooling of the head of the household in census tracts. Participants were considered hypertensive when the systolic blood pressure was ≥ 140 mmHg, diastolic ≥ 90 mmHg, or both. Additionally, the use of antihypertensive medication was also considered. Data were analyzed by using multilevel models of logistic and linear regression.

**RESULTS:**

The average age of the sample was 70.7 years and the average of systolic and diastolic blood pressure was 133.5 mmHg (SD = 20.5 mmHg) and 81.9 mmHg (SD = 12.5 mmHg), respectively. The systolic blood pressure was 4.46 mmHg (95%CI 1.00–7.92) higher and the chance of hypertension was 1.80 (95%CI 1.26–2.57) among those who lived in census tracts with lower level of schooling. When the use of antihypertensive medication was combined with blood pressure levels, none association was found between the outcome and the level of schooling of the census tract.

**CONCLUSIONS:**

Analytical models more robust (such as multilevel analysis) in Brazil are still little used, with a small number of articles published. Neighborhood socioeconomic status is associated with systolic blood pressure and the chance of hypertension, regardless of individual characteristics.

## INTRODUCTION

High systemic arterial blood pressure (BP) is a physiological change associated with a large number of chronic diseases, being one of the most important causes of cardiovascular problems and responsible for at least 45.0% of deaths due to heart disease and 51.0% of deaths due to stroke in 2008^25^.

The literature has evidence that individual demographic and socioeconomic characteristics are associated with high BP^25^. Most recently, studies have gone beyond individual factors and have shown that the place where people live is associated with several health outcomes, including BP^3^ ^,^ ^6^ ^,^ ^14^.

These studies have reported that neighborhood walkability, availability of recreational and leisure services, social cohesion, and perceived safety from crime are some determinants of hypertensive risk^6^ ^,^ ^14^. In addition, a growing – although still incipient and reduced – body of evidence suggests the influence of the neighborhood socioeconomic condition on hypertension^1^ ^,^ ^3^.

The hypotheses that relate the influence of the place of residence on health and more specifically on BP involve several aspects. Social features, in which neighborhoods with higher levels of crime, unsafe housing, and non-permanent jobs contribute to constant fear, stress, and victimization with biological impact and modification on the sympathetic nervous system activity and hormones such as cortisol^1^ ^,^ ^4^ ^,^ ^23^. Also, physical characteristics and infrastructure (as the availability of places for physical activity and food purchase) may cause elevation of BP. In addition, lower educational levels of the place of residence promotes the culture of knowledge and less healthy habits related to diet, physical activity, and use of health services, increasing the risk of developing hypertension^1^.

The analysis of the published literature on the influence of the neighborhood on BP has some limitations. First of all, most articles are carried out exclusively in high-income countries^6^ ^,^ ^12^ ^,^ ^14^ ^,^ ^21^. Moreover, the focus of most publications is on adults, with a few of them analyzing together – but not stratifying by age – data from older adults. This population group is of particular interest since BP tends to increase with age and the influence of the neighborhood context on BP among older people requires further investigation^6^ ^,^ ^9^ ^,^ ^10^ ^,^ ^15^ ^,^ ^21^. Finally, studies on hypertension in older people frequently use an indirect measure of BP, such as self-reported data^12^ ^,^ ^16^.

The aim of this study was to test if neighborhood socioeconomic status is associated with systolic BP (SBP) and hypertension in older adults.

## METHODS

A cross-sectional population-based study was carried out in Florianopolis, the capital of the Southern Brazilian state of Santa Catarina. The city had a Human Development Index of 0.847 in 2010, ranking third among all Brazilian cities, and the highest *per capita* household income mean among the country’s capitals (R$1,573.00)^a^ ^,^ ^b^. Furthermore, 11.5% of the population, according to the 2010 census, are older adults^c^.

The studied population consisted of non-institutionalized older people (60 years and older) of both sexes living in the urban area of the city between 2009 and 2010.

To calculate the sample size, the following parameters were considered: sampling error equal to 4 percentage points, expected prevalence of 50.0% (used to obtain the largest sample size), design effect of 2, and confidence interval of 95%. An over-sample of 20.0% was added considering estimated losses and 15.0% for control of confounders, resulting in a sample of 1,599 people. Several outcomes were investigated in this survey and the final sample after estimation for all of them was 1,911 people.

Cluster sampling was used to select the study sample. Census tracts (n = 420) were considered the primary sampling units, and the households were the secondary sampling units. The census tracts were stratified into deciles according to the average income of the head of the household. Eight census tracts were drawn in each decile to accommodate all income levels. As the last IBGE census took place nine years before the study, after the draw of the 80 census tracts, it was necessary to count all of the households in each sector. To reduce the coefficient of variation in the number of households per sector, making it more homogeneous, some of them were grouped and others were split, respecting the corresponding income decile. These changes were performed prior to data collection, when the supervisors of the study searched each of the selected tracts for research and counted all the occupied households, with the help of IBGE maps and images downloaded from Google Maps and Google Earth. This whole procedure followed the proposed rules by the IBGE and was accompanied by sampling specialists; also, this procedure was duly registered (allowing the reproducibility) and guided by clear and predefined criteria (avoiding subjectivity and bias). Finally, around 60 households were systematically drawn in each one of the 83 final census tracts.

The field team consisted of 20 female interviewers. All of them were educated above high school level. The interviewers were trained before the fieldwork and participated in the pilot study, carried out with older people living in census tracts not included in the sample.

Interviews were conducted using personal digital assistants (PDA) between the second half of 2009 and the first half of 2010. Data were collected via individual interviews using a structured questionnaire.

All older residents in the selected households were invited to participate in the study. Losses were considered when it was not possible to carry out the interviews after four attempts (including at night and on weekends).

To ensure the quality of the data, data consistency was checked weekly and inconsistencies were corrected. For quality control purposes, we randomly selected 10.0% of the interviews for the application of a brief questionnaire with key questions via telephone.

In this study, hypertension was assessed in two ways. First, older adults were considered hypertensive if they had SBP ≥ 140 mmHg or diastolic blood pressure (DBP) ≥ 90 mmHg^2^, or both. Second, those who reported using antihypertensive medication or whose SBP was ≥ 140 mmHg or DBP was ≥ 90 mmHg were considered hypertensive.

To measure BP, the participant had been at rest for at least five minutes, in a calm environment, with an empty bladder, had not performed exercise, smoked or ingested food, coffee or alcoholic beverages 60 to 90 minutes before the interview. Finally, the interviewee could not talk during the measurement. Systolic and diastolic BP were measured once on each arm, using electronic digital reading devices (Techline Z-40 model) (Techline, Taiwan, China). For the analysis, the means of SBP and DPB were calculated for each arm and the final pressure level average of the arm with the highest value was considered.

All subjects were asked about medications taken in the previous 30 days. Active substances and indication of use of each medicine were identified and analyzed according to the Anatomical Therapeutic Chemical (ATC), established by the WHO Collaborating Center for Drug Statistics Methodology^24^.

The contextual variable used was the mean years of schooling of the head of the household in each census tract. Data were collected from the 2000 Brazilian census. The variable was divided into three categories: ≤ 8, 9-11, and ≥ 12 years of schooling.

Individual demographic and socioeconomic explanatory variables were gender, age (60-69 years; 70 or more), education (≤ 8; 9-11; ≥ 12 years of study) and equalized household income (divided into tertiles). These variables were collected via interviews. Gender was seen by the interviewer, age was calculated from the date of birth, education was obtained in full years of study, and equalized *per capita* household income was calculated by dividing the household income by the number of dependent household income squared.

Data analysis was conducted in Stata 13.1 statistical software (Stata Corp., College Station, USA) and included the sample description, the distribution of mean SBP, odds of hypertension, and the 95% confidence intervals (95%CI), according to contextual and individual variables.

We carried out multilevel logistic and linear regression, since the outcome (hypertension) was analyzed as dichotomous (two different outcomes, one considering only SBP and DBP and the other considering medication use) and continuous variables (analyzing the mean of SBP). The analyses followed a theoretical model, in which each variable was included in one of the different models presented after literature review. Analyses of variance were observed in the different models to verify whether they were explaining the variation of the outcome well. In both analyses, a hierarchical model with two levels, the individual (level 1) and contextual income (level 2), was employed. The multivariate analysis was fitted with four models using a forward procedure of regression analyses: empty model (without covariates), model 1 (included only contextual education), model 2 (demographic variables including gender and age), and model 3 (included the individual income and education variables).

The full multilevel model used in the linear regression was:


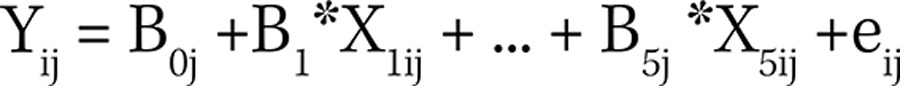


Where B_0j_ = γ_00_ + γ _0j_*W_j_ + u_0j_; B_ij_ = γ _i0_+ γ _i1_*W_j_ + u_1j_

In the equation, Yi is the outcome, in this case the average SBP, and B_0_ is the intercept. The explanatory variables at the individual level are indicated by the variables Xij and the contextual level is denoted by Wj. The variable “u” refers to the random effect and “e” refers to the residues of the equation.

The intra-class correlation (ICC) of the multilevel linear regression was calculated as follows: level 2 variance / (level 2 variance + level 1 variance).

In the multilevel logistic regression model, the ICC was defined as: level 2 variance / level 2 variance + (π^2/3). In both cases, the ICC provides an estimate of the total variance in BP or hypertension prevalence that can be attributed to differences among the neighborhoods. In linear regression analyses, the intercept with the mean of BP and fixed and random effects were calculated and presented for each model. In logistic regression analyses, random effects were presented. Confidence intervals (95%CI) are reported in the tables and effect measurements were compared with their reference categories. Interaction between individual and contextual socioeconomic variables was performed.

Regarding the study power, the calculation in multilevel analysis is complex and still is object of study. Snijders^20^ points out that the number of sectors is more important than the number of individuals in each of them. In the study EpiFloripa, we have 83 census tracts, numbering more than 50, considered to be acceptable to estimate different parameters in multilevel analyses. Also, it was estimated the effect design for the cluster samples to be small, ranging from 1.48 to 2.08.

The project was approved by the Ethics Committee of Research with Human Beings of the Universidade Federal de Santa Catarina (Process 352/2008, December 23, 2008). Respondents were asked to sign an informed consent form before the interview.

## RESULTS

The final sample consisted of 1,705 older adults (response rate of 89.2%). Most participants were women and reported having eight years or less of study. The average age was 70.7 years (SD = 8.0 years). Regarding the area-level education, the average years of study of the householder was between nine and eleven in most census tracts (43.2%). The description of the whole sample can be seen in Table 1.

**Table 1:** Characteristics of the older population studied. Florianopolis, SC, Southern Brazil, 2009-2010.

Characteristic			Systolic BP (mmHg)		Diastolic BP (mmHg)		High BP^a^		High BP^b^	
				
	n	%	mean	SD	mean	SD	%	95%CI	%	95%CI
Individual level										
Age (years)										
60-69	854	50.1	137.3	19.6	83.3	12.0	64.1	60.0–68.2	76.4	72.9–79.6
≥ 70	851	49.9	139.8	21.3	80.4	12.8	64.3	60.1–68.4	85.2	82.6–87.6
Gender										
Male	616	36.1	140.3	20.5	84.5	12.9	68.8	64.1–73.2	84.1	80.5–87.1
Female	1,089	63.9	137.5	20.4	80.4	12.0	61.6	57.8–65.3	78.9	76.0–81.6
Education (years of schooling)										
≥ 12	394	23.3	137.5	18.3	82.8	11.7	65.4	59.0–71.1	79.6	74.6–83.9
9-11	234	13.8	135.6	17.9	81.4	11.9	58.5	49.7–66.7	75.6	68.7–81.8
≤ 8	1,066	62.9	139.6	21.8	81.6	12.9	65.1	61.5–68.8	82.5	79.7–84.9
Equalized household income										
Higher tertile	569	33.4	139.7	22.5	81.5	13.3	63.5	58.1–68.4	80.9	76.8–84.3
Intermediary tertile	568	33.3	138.9	19.5	82.3	11.5	66.8	61.8–71.6	82.1	78.4–85.6
Lower tertile	568	33.3	137.0	19.3	81.9	12.7	62.4	57.0–67.4	79.3	75.3–83.0
Contextual level (census tracts, n = 83)										
Area-level education (mean years of schooling)										
≥ 12	488	28.6	136.0	18.5	80.7	12.3	57.6	51.7–63.6	78.3	73.9–82.4
9-11	736	43.2	139.0	20.3	82.6	12.1	65.3	60.9–69.7	80.9	77.3–83.9
≤ 8	481	28.2	140.4	22.4	82.0	13.2	69.3	64.0–74.2	83.2	79.0–86.6

BP: blood pressure

^a^ High blood pressure: systolic blood pressure ≥ 140 or diastolic blood pressure ≥ 90 mmHg.

^b^ High blood pressure: systolic blood pressure ≥ 140 or diastolic blood pressure ≥ 90 mmHg or currently use of anti-hypertensive medications.

The average SBP and DBP was 138.5 (SD = 20.5) mmHg and 81.9 (SD = 12.5) mmHg, respectively. Higher prevalence of hypertension was found in males (68.8% and 84.1%, depending on the diagnostic criteria) and no difference in the outcome was found according to individual schooling and income. The prevalence of hypertension was higher among those who lived in areas with lower levels of schooling (Table 1).

Table 2 shows the results of the multilevel linear regression models. It was observed in the fully adjusted model that the between-group variance corresponded to 2.7% of the total variance. Even after adjusting for individual characteristics, area-level education was inversely associated with the average values of BP. In the fully adjusted model, the SBP was 3.97 mmHg (95%CI 0.50–7.44) higher among residents of low-education neighborhoods.

**Table 2:** Multivariate models of multilevel linear regression for systolic blood pressure in population ≥ 60 years old, city of Florianopolis, SC, Southern Brazil, 2009-2010.

Variable	Empty model	Model 1	Model 2	Model 3
			
	Coefficient (95%CI)	Coefficient (95%CI)	Coefficient (95%CI)	Coefficient (95%CI)
Fixed effects				
Intercept (mean of BP)	138.5 (137.3–139.8)	136.0 (133.8–138.2)	136.2 (133.4–139.0)	136.3 (133.2–139.4)
Contextual level (census tracts, n = 83)				
Area-level education (mean years of schooling)				
≥ 12		Reference	Reference	Reference
9-11		3.02 (0.13–5.90)*	3.13 (0.21–6.04)*	2.74 (-0.29–5.76)
≤ 8		4.45 (1.25–7.63)*	4.63 (1.40–7.85)*	3.97 (0.50–7.44)*
Age (years)				
60-69			Reference	Reference
≥ 70			2.92 (0.97–4.87)*	2.75 (0.75–4.75)*
Gender				
Male			Reference	Reference
Female			-2.73 (-4.75–-0.71)*	-2.74 (-4.80–-0.68)*
Education (years of schooling)				
≥ 12				Reference
9-11				-2.21 (-5.61–1,19)
0-8				0.18 (-2.71–3.05)
Equalized household income				
Higher tertile				Reference
Intermediary tertile				0.71 (-1.90–3.31)
Lower tertile				0.99 (-1.76–3.75)
Random effects				
Level-two variance	13.4	10.8	11.6	11.3
Level-one variance	408.4	408.4	404.8	406.4
ICC	3.18	2.58	2.79	2.70

BP: blood pressure; ICC: intra-class correlation

* p < 0.05.

Logistic regression showed similar results, with an increase in the odds of hypertension in the categories with lower area-level education (Table 3). These odds were 1.76 times higher (95%CI 1.23–2.51) among those who lived in low-education areas. By analyzing the values of ICC, it can be observed that the variance between groups represented 3.06% of the total variable in the final analyzed model.

**Table 3:** Multivariate models of multilevel logistic regression for high blood pressure in population ≥ 60 years old, city of Florianopolis, SC, Southern Brazil, 2009-2010.

Variable	High blood pressure^a^				High blood pressure^b^			
	
	Empty model	Model 1	Model 2	Model 3	Empty model	Model 1	Model 2	Model 3
							
	OR (95%CI)	OR (95%CI)	OR (95%CI)	OR (95%CI)	OR (95%CI)	OR (95%CI)	OR (95%CI)	OR (95%CI)
Fixed effects								
Contextual level (census tracts, n = 83)								
Area-level education (mean years of schooling)								
≥ 12		Reference	Reference	Reference		Reference	Reference	Reference
9-11		1.39 (1.04–1.86)^c^	1.38 (1.03–1.85)^c^	1.41 (1.04–1.92)^c^		1.17 (0.85–1.60)	1.21 (0.88–1.66)	1.20 (0.85–1.68)
≤ 8		1.67 (1.21–2.31)^c^	1.67 (1.20–2.32)^c^	1.76 (1.23–2.51)^c^		1.37 (0.97–1.97)	1.45 (1.02–2.08)^c^	1.42 (0.95–2.12)
Age (years)								
60-69			Reference	Reference			Reference	Reference
≥ 70			1.06 (0.87–1.31)	1.09 (0.88–1.35)			1.86 (1.44–2.40)^c^	1.86 (1.43–2.41)^c^
Gender								
Male			Reference	Reference			Reference	Reference
Female			0.72 (0.58–0.90)^c^	0.75 (0.60–0.94)^c^			0.69 (0.53–0.90)^c^	0.71 (0.54–0.93)^c^
Education (years of schooling)								
≥ 12				Reference				Reference
9-11				0.84 (0.62–1.14)				0.73 (0.48–1.10)
0-8				0.71 (0.49–1,01)				1.03 (0.71–1.47)
Equalized household income								
Higher tertile				Reference				Reference
Intermediary tertile				1.10 (0.84–1.46)				1.07 (0.76–1.48)
Lower tertile				0.93 (0.70–1.24)				0.91 (0.64–1.29)
Random effects								
Level-two variance (SE)	0.13 (0.05)	0.09 (0.05)	0.10 (0.05)	0.10 (0.05)	0.07 (0.06)	0.06 (0.05)	0.06 (0.06)	0.07 (0.06)
ICC (%)	4.01	2.29	2.97	3.06	2.14	1.80	1.80	2.25

SE: standard error; ICC: intra-class correlation

^a^ High blood pressure: systolic blood pressure ≥ 140 or diastolic blood pressure ≥ 90 mmHg.

^b^ High blood pressure: systolic blood pressure ≥ 140 or diastolic blood pressure ≥ 90 mmHg or currently use of anti-hypertensive medications.

^c^ p < 0.05.

In Table 3, hypertension is also analyzed as the combination of use of antihypertensive medication and SBP ≥ 140 and/or DBP ≥ 90 mmHg. Using this definition of hypertension, no association between area-level education and the outcome was found. Furthermore, the analyses did not show interaction between individual and contextual socioeconomic variables.

## DISCUSSION

The results of this study indicate that the area-level education is an important factor associated with the mean SBP and the odds of hypertension among older people regardless of individual characteristics. In both analyses, a dose-response association was found.

These findings are consistent with other studies carried out previously in countries such as Canada^10^ ^,^ ^12^, China^9^, United States^6^ ^,^ ^14^, Brazil^8^, and France^21^, where people living in neighborhoods with lower housing conditions had higher BP. A different result was found by Dragano et al.^5^, with data from the Czech Republic and Germany, where no association between neighborhood-level socioeconomic status and hypertension was found.

The higher prevalence of hypertension and the higher mean of SBP in people who live in lower socioeconomic status neighborhoods may be explained by different paths. One aspect is related to the lower social cohesion, greater sense of criminal victimization, fear and more stressful situations experienced among people living in these neighborhoods^3^ ^,^ ^15^. Besides this potential consequence in behavior, stress may have a biological impact. Studies reported a lower rate of decline in cortisol during the day in people living in more violent neighborhoods, for example^4^.

Other aspect is the physical structure of the neighborhood and the access to public and private facilities that are beneficial to health, such as healthy food outlets and gyms. People living in lower socioeconomic neighborhoods have higher availability of fast food outlets and less healthy food options^7^. Besides, it is more difficult to perform physical activity in these neighborhoods, either due to the lack of appropriate places or because of the feeling of insecurity to leave the house^19^. As a result, obesity and low physical activity, known risk factors for high BP, are reported to be more frequent in lower socioeconomic neighborhoods.

Pharmacological and non-pharmacological treatments are capable of improving the prognosis and the quality of life of hypertensive people^11^, therefore the access to a good quality health service is important for the control of arterial BP. Nonetheless, inequalities have been reported in the access to the health services depending on the socioeconomic status of the neighborhoods^26^.

In this study, although the mean SBP and hypertension defined by the clinical values of BP were associated with area-level education, no association was found when the definition of hypertension included the use of medicines. A possible explanation may be related to the different quality of information. While the clinical measure of BP was obtained using high-quality equipment and well trained professionals, the use of medicines was self-reported. Although the use of self-reported measures seems to be satisfactory^22^, studies in Brazil^13^ ^,^ ^17^ indicate that this type of measure should be used with caution according to age and socioeconomic status, as the prevalence of hypertension is underestimated when using these self-reported measures in this population. Another explanation may be the better control of BP levels by residents in neighborhoods with higher education, with better treatment, causing this difference when the medicines were included in the analysis. Furthermore, another reason to this difference could be a lack of power of the study to find significant associations when the difference between groups is lower.

Limitations of this study should be considered. First, the cross-sectional design does not support causal inferences. We also did not investigate the length of residence in the neighborhood, which could be important to quantify the exposure to the environmental characteristics. Furthermore, all older people living in the selected households were potential participants of the study and, consequently, individuals sharing the same behaviors and genetically related were sampled. The use of Census 2000 data, collected nine years before this study, was not considered a limitation, as the effects of neighborhood on health outcomes act in a chronic form. There is a minimum latency period between neighborhood characteristics and their effects on BP, a fact that could not be well measured when using the 2010 Census data^18^.

However, this study presents several strengths. We analyzed a representative sample and used small – and consequently more homogeneous – areas of the city. The study also presented a high response rate that was similar in all the income deciles of the census tracts. Also, the outcome was measured according to international standards. Furthermore, we believe this is the first study that investigated the influence of neighborhood socioeconomic status on hypertension in Latin American older people and one of the few in the world.

Moreover, it can be noted that more robust analytical models (such as multilevel analysis) only recently (around two decades) was incorporated into epidemiological studies. Particularly in Brazil, the production of knowledge with this approach is still new and a small number of articles were published^8^.

Considering that the prevalence of hypertension among older people has remained at high levels for the past 30 years, it is possible that interventions oriented strictly to the individual level may not have had sufficient effect on the control of this comorbidity^17^. In so far as the area-level education has an inverse relationship with the risk for high BP in the older participants, the results of this study may contribute to the identification of the population at risk for hypertension and planning actions to improve the quality of life of these people. Thus, to control hypertension, policy makers should consider not only the individual characteristic but the socioeconomic neighborhood context as well.

## Footnotes

a Programa das Nações Unidas para o Desenvolvimento. Atlas do desenvolvimento humano no Brasil 201: com dados dos censos 1991, 2000 e 2010 [cited 2014 May 25]. Available from: http://www.pnud.org.br/atlas/ranking/Ranking-IDHM-Municipios-2010.aspx

b Exchange rate average in July 2010: BRL 1.00 = USD 1.77

c Instituto Brasileiro de Geografia e Estatística. Características da população e dos domicílios: resultados do universo. Rio de Janeiro: IBGE; 2011 [cited 2015 Oct 19]. Available from: http://censo2010.ibge.gov.br/resultados
